# Autoimmune Hemolytic Anemia in the Pediatric Age Group: The Egyptian Experience

**DOI:** 10.1007/s00277-023-05230-5

**Published:** 2023-04-24

**Authors:** Amina Abdel-Salam, Sherifa Tarek Bassiouni, Alaa Magdi Goher, Eman Shafik Shafie

**Affiliations:** 1 Deparment of Pediatrics, Faculty of Medicine, Cairo University, Cairo, Egypt; 2 Department of Clinical and Chemical Pathology, Faculty of Medicine, Cairo University, Cairo, Egypt

**Keywords:** Autoimmune hemolytic anemia, Children, Egypt

## Abstract

Autoimmune hemolytic anemia (AIHA) is a common disease entity among adults; however, it is rare among the pediatric age group. Evidence is scarce regarding pediatric AIHA in the literature. The objective of this study is to assess the frequency of AIHA and describe the clinical and laboratory characteristics and treatment outcomes of a cohort of children with AIHA in Egypt. A retrospective study was conducted on 50 children with AIHA who were registered and followed up at the New Children’s Hospital in Cairo, Egypt, between January 2010 and January 2021. The study group comprised 60% females and 40% males. Their median age was 8.25 years. All patients showed low hemoglobin levels with a mean of 5.40 ± 1.34 g/dl and a median reticulocyte count of 10 (IQR: 8–15). Twelve (24%) patients were diagnosed with Evans syndrome, and a positive Coombs test was detected in 46 patients (92%). The frequency of primary AIHA was 40%, whereas it was 60% for secondary AIHA. The first line of therapy for acute attacks was high-dose IV steroids which responded well in 38 (76%) patients. Secondary AIHA was more common among our children (60%). AIHA is more prevalent in females (60%). The clinical and laboratory characteristics matched previous reports.

## Introduction

Autoimmune hemolytic anemia (AIHA) is a rare blood disorder characterized by autoantibodies that bind to the erythrocyte surface membrane and lead to premature red cell destruction. Though it can occur at any age, it is scarce in infancy and childhood, and its estimated incidence is 0.2 per 100,000/year [1]. The diagnosis is based classically on the clinical and biological signs of hemolysis and the direct antiglobulin test (direct Coombs test) [2, 3].

Most children suffer from pallor, jaundice, tiredness, or dark urine. Less commonly, there will be fever or abdominal pain, and 3% of children presented with collapse, coma, or acute renal insufficiency due to sudden and severe anemia [4]. Positive Coombs test (DAT) is the diagnostic hallmark of AIHA; however, it may be absent in some pediatric cases with a frequency ranging from 6 to 23% [5].

Classification of AIHA is pathophysiologically based and divides AIHA into warm, mixed, or cold-reactive subtypes. This thermal-based classification is based on the optimal autoantibody-RBC reactivity temperatures. AIHA is further subcategorized into idiopathic and secondary, associated with several underlying infectious, neoplastic, and autoimmune disorders [6].

Corticosteroids are used as first-line therapy of warm AIHA, and 70–80% of patients show improvement after 3 weeks of treatment; intravenous immunoglobulins may be indicated as adjunctive therapy to steroids in more severe cases [7].

For refractory and relapsed cases, the choice may be between splenectomy and rituximab, which is becoming the preferred second-line treatment [8]. Some authors prefer rituximab to splenectomy as a second-line treatment for children with AIHA due to the higher risk of infective complications following this practice in younger children [9]. In this study, we aimed to assess the frequency, clinical features, and treatment outcomes of primary and secondary AIHA among Egyptian children.

## Patients and methods

This retrospective study included fifty AIHA patients registered at hematology and/or rheumatology clinics at New Children’s Hospital, Cairo, Egypt, from January 2010 to January 2021. Patients were 30 (60%) females and 20 (40%) males with female sex predominance; m/f ratio is 2:3. Their ages ranged from 1.1 to 14 years with a median of 8.25 years. Age at diagnosis ranged from 0.9 to 12 years with a median of 5.5 years, and they were diagnosed as AIHA based on conventional clinical and hematologic criteria [10]. Patients with AIHA are further subcategorized into idiopathic and secondary depending on a recognized underlying cause, such as immunodeficiency, infections, medications, or malignancy [6]. Primary and secondary AIHA of both sexes aged 18 years old or under were included in the study. Patients with laboratory evidence of other hemolytic disorders, e.g., hereditary spherocytosis; patients younger than 3 months; those with laboratory evidence of autoimmune lymphoproliferative syndrome; and patients with incomplete follow-up information at the time of data collection were excluded. Informed consent was obtained from the patients or their legal guardians before enrollment in the study. Data confidentiality and informants’ identity were maintained throughout the study and were coded and accessed by the investigators only. The Ethical Committee of Cairo University approved the study protocol with reference number MS-432-2021, according to the Institutional Committee for the Protection of Human Subjects and following the 18th World Medical Assembly, Helsinki, Finland.

Data were retrieved by reviewing medical records and directly interviewing all the patients and their legal guardians. Clinical and laboratory data were recorded at the first presentation (baseline). The collected clinical data included demographics, preceding illnesses, symptoms, blood transfusion history, and drug therapy. The collected laboratory studies included complete blood count, reticulocyte count, and DAT (Coombs test). Virology screening for Epstein-Barr virus, hepatitis, HIV, cytomegalovirus , and the systemic lupus erythematosus (SLE) panel (C3, C4, ANA, and anti dsDNA) was carried out for all patients once diagnosed as AIHA, and data were collected. Patients suspected of having autoimmune hepatitis were investigated for ANA, AMA, ASMA, and LKMA. Bone marrow examination was conducted for patients with associated thrombocytopenia to exclude underlying malignancy or lymphoproliferative disorder. Results of any additional diagnostic tests were collected (e.g., a renal biopsy was performed for SLE patients diagnosed).

### Follow-up data

Patients’ follow-up information, including clinical and laboratory response data, were obtained from outpatient records. The last available laboratory results were collected, including complete blood count, reticulocyte count, and DAT. Any relapses data were collected.

### Treatment data

#### First-line treatment

As per our local treatment protocol, this line includes corticosteroids with or without intravenous immunoglobulin (IVIG):

I. Corticosteroids:

○ For the most severely affected patients, such as those with rapidly evolving and severe hemolysis, pulse high-dose methylprednisolone, a dose of 30 mg/kg with a maximum dose of 1 g once daily for 3 days, was applied [11, 12].

○ Children with mild or moderate anemia (e.g., hemoglobin ≥7 to 8 g/dL) and appropriate reticulocytosis can be initially treated with oral prednisone 2 mg/kg per day in two or three divided doses [13].

○ Oral prednisone, 2 mg/kg per day, was used as the initial maintenance treatment in all patients upon discharge from the hospital. Withdrawal of prednisone was carried out during the follow-up visits. Adding a second-line/third-line therapy was guided by the patient’s response.

II. **Intravenous immunoglobulin IVIG** was combined with steroids in severe and refractory cases (i.e., patients with rapid hemolysis and very severe anemia or complex cases such as Evans syndrome) at 1g/kg/day for 2 days.
III. Plasmapheresis was considered in patients who showed ongoing hemolysis after receiving combined IVIG and steroids.

#### Second-line therapy

It was indicated in patients with relapsed AIHA and those with resistant AIHA who showed initial poor response to steroids. Second-line therapy includes the following:

I. **Azathioprine**: An immunosuppressive agent that primarily affects T lymphocyte helper function, thereby diminishing autoantibody synthesis and exerting a steroid-sparing effect. It was given at 3–4 mg/kg/day.
II. **Cyclosporine A**: Another immunosuppressive agent that primarily affects T lymphocytes; it should only be used in children with refractory AIHA. The recommended starting oral dose is 1 to 3 mg/kg daily, divided into two doses 12 h apart [14].
III. Mycophenolate **mofetil (MMF)**: This immunosuppressive agent has been used in patients with relapsed, resistant AIHA, including those who have had a solid organ or stem cell transplant or who have Evans syndrome, acquired pure red cell aplasia, and/or ALPS [15, 16]. The recommended dose is 600 mg/m^2^ orally divided every 12 h.

#### Third-line therapy

It was indicated in children with refractory AIHA who have failed with second-line therapy [13].

I. **Splenectomy**: It is typically reserved for patients who fail medical therapy [17].
II. **Rituximab**: Rituximab is an anti-CD20 monoclonal antibody used for children with AIHA who are refractory to or dependent on glucocorticoids [18].
III. **Cyclophosphamide**: It has been reported in a few refractory cases. The recommended dose is 500 mg/m^2^ intravenously every 4 weeks for three doses.
IV. **Response criteria were classified as follows**:
  ○ **Complete remission**: Clinical symptoms disappeared, with normalized erythrocyte count, hemoglobin level, reticulocyte count, bilirubin level, and negative DAT.
  ○ **Partial response**: Clinical symptoms controlled, with hemoglobin level more than 8 g/dL, reticulocyte count less than 5%, bilirubin less than 3.4 mg/dL, as well as a negative or reducing titer of DAT.
  ○ **No response:** The case was assigned as non-responsive when anemia or the persistence of symptoms of hemolysis and the laboratory criteria did not achieve a partial response [19].

### Statistical methods

Data were coded and entered using the statistical package SPSS version 21, IBM, USA. Data were summarized using the range, mean, standard deviation (SD), median, frequencies (number of cases), and percentages when appropriate. A comparison of numerical variables between the study groups was performed using a *t*-test for independent samples when comparing two groups. For comparing categorical data, a chi-square *χ*^2^ test was performed. An exact test was used when the expected frequency was less than 5. Repeated measures ANOVA and Friedman tests were used to compare laboratory data at three different time points. Results were considered statistically significant at a *p*-value of less than or equal to 0.05.

The sample size was calculated using the *GPower®* version 3.1.9.4 program [20]. The statistical calculator is based on a 95% confidence interval, and the power of the study is 90% with α error of 5%. According to a previous study [5], the sample size was calculated, and a minimal sample size of 30 cases was enough to find a difference.

## Results

Most cases were of non-consanguineous marriages 29 (58%) and 2 (4%) patients had similar diseases in the family. The most common presenting symptom was pallor 49 (98%), followed by dark urine 41 (82%). Other features are listed in Table 1. Laboratory-documented viral infections, drug history, and comorbidities preceding the onset are shown in Table 1.

**Table 1:** The frequency of occurrence of different symptoms of 50 children with AIHA

Presenting symptoms	No.	%
Pallor	49	98.0%
Jaundice	38	76.0%
Dark urine	41	82.0%
Fever	10	20.0%
Muscle pain	9	18.0%
Headache	8	16.0%
Nausea/vomiting	2	4.0%
Bleeding from any orifices	7	14.0%
Viral infection preceding the attack	No.	%
CMV	9	18.0%
EBV	6	12.0%
Hepatitis	6	12.0%
Rubella	1	2.0%
Drug history		
Penicillins	3	6.0%
Cephalosporins	1	2.0%
Acetaminophen	1	2.0%
Ibuprofen	1	2.0%

**Fig. 1:**
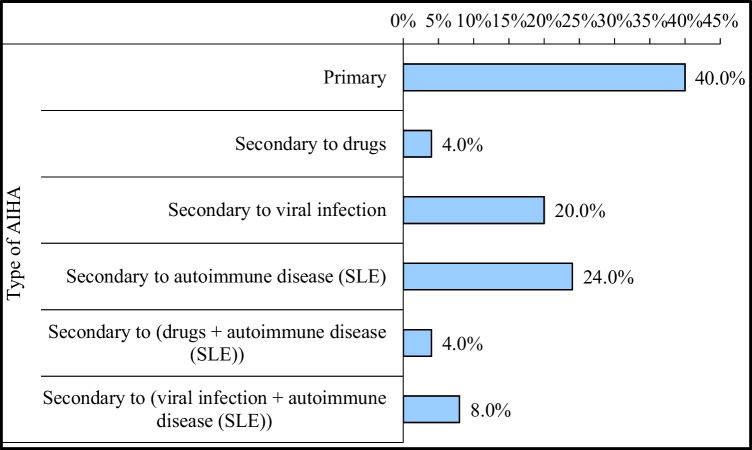
The underlying etiologies in secondary AIHA (*n*=30)

Patients were divided into two subgroups according to their classification into primary (idiopathic) AIHA (*n*=20) and secondary AIHA (*n*=30). The underlying etiology in secondary AIHA is illustrated in Fig. 1.

### Laboratory investigation at 1st presentation

Labs at the first presentation of 50 AIHA cases are shown in Table 2. Hemoglobin level showed a mean of 5.40 ± SD 1.34 g/dl, mean platelets 240.92 ± SD 131.79 (10^3^/cc), mean TLC 8.73 ± SD 4.13 (10^3^/cc), and median (IQR) of retics was 10 (8–15) with a range of 4–23. Twelve (24%) patients have associated thrombocytopenia at first presentation and were diagnosed with Evans syndrome. Among 50 patients with AIHA, 46 (92%) cases had Coombs test positive, and the rest of the cases, 4 (8%), showed negative tests.

**Table 2:** Laboratory investigation of 50 patients at first presentation

Labs at presentation	No. = 50	
	Mean ± SD	Range
Hb (gm/dl)	5.40 ± 1.34	2.5–8
MCV (fl)	72.49 ± 10.01	45–100
MCH (pg)	26.08 ± 4.62	17–38
Hct (%)	20.20 ± 5.38	9–33
Platelets (10^3^/cc)	240.92 ± 131.79	6–558
TLC (10^3^/cc)	8.73 ± 4.13	2–25
Retics (%)	10 (8 – 15)	4–23
Lupus profile	No.	%
C3		
Normal	31	62.0%
Consumed	19	38.0%
C4		
Normal	32	64.0%
Consumed	18	36.0%
ANA		
Negative	29	58.0%
Positive	21	42.0%
Anti-dsDNA		
Negative	32	64.0%
Positive	18	36.0%

All cases in our study were screened for rheumatological diseases, especially SLE. The results are illustrated in Table 2. Regarding the lupus profile, C3 was consumed in 19 (38%) cases, C4 in 18 (36%) cases, ANA positive in 21 (42%) cases, and anti-dsDNA positive in 18 (36%) cases.

*Hb* hemoglobin, *MCV* mean corpuscular volume, *MCH* mean corpuscular hemoglobin, *Hct* hematocrit, *TLC* total leucocyte count, *C3* complement component 3, *C4* complement component 4, *ANA* anti-nuclear antibody, *Anti-dsDNA* anti double-stranded DNA

*Other investigations* were performed for special groups that had another association with AIHA. In twelve patients (24%) who had thrombocytopenia, BMA was conducted and revealed hypercellular marrow in 8 (16%) and normocellular in 4 (8%). Renal biopsy was performed for 10 (20%) patients who were diagnosed with SLE, and there were lupus nephritis class 4 in 6 (12%) patients, lupus nephritis class 2 and 3 were equal, and each was found in 2 (4%) patients. ASMA, AMA, and LKMA profile was performed in 4 (8%) patients suspected of having autoimmune hepatitis, and it was positive in the other 2 (4%) patients. HCV-PCR was performed in 3 (6%) patients, and it was positive in 2 (4%) and negative in 1 (2%) patient. CMV-PCR was conducted in 6 (12%) patients, and all were negative. Fibroscan was performed for 2 (4%) patients with hepatitis associated with AIHA and showed fibrosis grade 1 in both patients.

### Treatment at 1st presentation

Blood transfusion was required in all patients with a median transfusion frequency of 2 (IQR: 1–2; range 1–8). The recurrent mismatch was found in 44 (88%) cases. All children (*n* = 50) received IV steroids as the first line of treatment with a mean duration of 11.74 (± 5.77) days (range: 3 to 20 days). Thirty-eight (76%) patients were responders and did not show hemolysis afterward. Twelve (24%) showed ongoing hemolysis after the first line. They were rescued with IVIG (*n* =11) and plasmapheresis (*n* = 5).

Maintenance treatment after the first line included steroids in 38 (76%) patients. Mycophenolate mofetil (MMF) was added in 11 (22%) patients, and a combination of oral steroids, Mycophenolate mofetil, and Prograf (tacrolimus) in 1 (2%) patient.

Forty-one (82%) patients showed a good response to treatment, and 9 (18%) patients showed frequent relapses during the withdrawal of steroids.

A comparison of laboratory parameters at presentation, after first-line treatment, and at follow-up is presented in Table 3. Mean hemoglobin level and mean platelet count were significantly increased. Median reticulocyte counts dramatically decreased (*p* < 0.05) (Table 3).

**Table 3:** Laboratory parameters at presentation, after first-line treatment, and at follow-up (*n* = 50)

Labs	At presentation	After 1st line	Last follow-up	*p*-value	
	No. = 50	No. = 50	No. = 50		
Hb (gm/dl)	Mean±SD	5.40 ± 1.34	10.07 ± 1.59	11.44 ± 1.32	<0.05*
	Range	2.5–8	7.5–14.5	9–14.5	
PLT (10^3^/cc)	Mean±SD	240.92 ± 131.79	280.06 ± 120.41	328.36 ± 114.83	<0.05*
	Range	6–558	45–546	71–750	
TLC (10^3^/cc)	Mean±SD	8.73 ± 4.13	10.53 ± 3.13	11.63 ± 3.02	>0.05
	Range	2–25	5–20	5–18	
Reticulocyte count (%)	Median (IQR)	10 (8–15)	2 (1–3)	0.8 (0.5–1.5)	<0.05*
	Range	4–23	0.5–9	0.4–7	

*Hb* hemoglobin, *MCV* mean corpuscular volume, *TLC* total leucocyte count, *PLT* platelet count

*p-value less than or equal to 0.05 is considered statistically significant

### Comparison of primary and secondary AIHA

Children with primary AIHA were predominantly males (*p* < 0.0001); they were younger (*p* < 0.0001) and had a lower age at diagnosis (*p* = 0.001). Details are illustrated in Table 4. All primary AIHA patients had jaundice and dark urine at presentation, while jaundice was seen in 60% and dark urine in 70% of secondary AIHA patients (*p* = 0.001 and 0.007, respectively). Associated symptoms, including fever, muscle pain, and headache, were reported only among the secondary AIHA (33.3%, 30%, and 26.7%, respectively). Weakness and tiredness were more prevalent in the secondary type (66.7% vs. 35%, *p* = 0.05). Five patients with primary AIHA (5/20, 25%) had associated thrombocytopenia versus 6 (6/30, 20%) with secondary AIHA (*p* = 0.676). Other laboratory parameters at presentation were comparable between primary and secondary AIHA patients.

**Table 4:** Differences in primary (*n* = 20) and secondary AIHA (*n* = 30)

		Type of AIHA		*p*-value	Sig.
		Primary AIHA	Secondary AIHA		
		No. = 20	No. = 30		
Gender					
Male		14 (70.0%)	6 (20.0%)	<0.0001	HS
Female		6 (30.0%)	24 (80.0%)		
Age (years)	Median (IQR)	4.25 (2.3–6)	11 (8–12)	<0.0001	HS
Age at diagnosis (years)	Median (IQR)	3.55 (1.65–5)	9.5 (3.6–11)	0.001	HS
Duration of follow-up (months)	Median (IQR)	11 (6–12)	12 (6–24)	0.145	NS

**Fig. 2:**
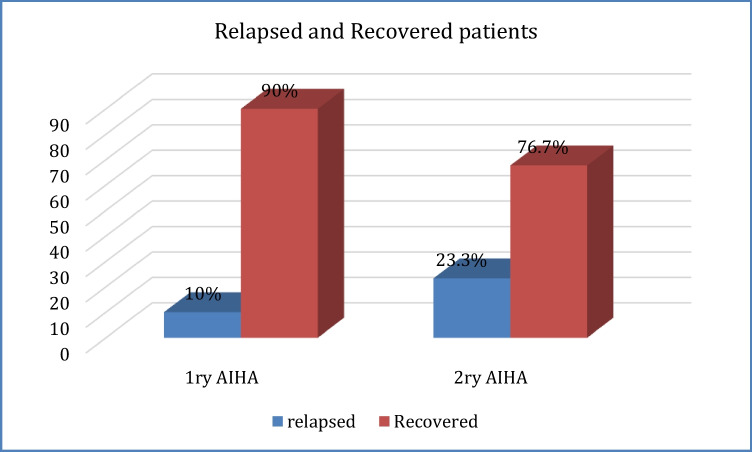
Comparison of the relapse rate among patients with primary and secondary AIHA

During the withdrawal of steroids, the relapse rate among patients with primary AIHA was 10% (2 out of 20 patients) versus 23.3% in secondary AIHA (*p*=0.2), as illustrated in Fig. 2.

### Patients with relapses (n=9)

The median age of non-responders was 9 years (IQR: 8.5–11.8). Their median duration of follow-up was 42 months (IQR: 18–66). Five (55.6%) had a history of positive consanguinity, and 1 (11.1%) had a similar condition in his family. Details of clinical and laboratory data are illustrated in Table 5.

**Table 5:** Clinical and laboratory data of patients with relapses (*n* = 9)

	No. (*n*=9)	%
Other systems affection	4	44.4%
Neurological Rheumatologic disease Gastrointestinal tract	2	22.2%
	1	11.1%
	1	11.1%
Recurrent mismatch	9	100.0%
Coombs test		
Negative	0	100.0%
Positive	9	0.0%
Type of AIHA		
Primary	2	22.2%
Secondary	7	77.8%
Secondary to viral infection	6	66.7%
Secondary to autoimmune disease (SLE)	1	11.1%
Associated thrombocytopenia		
No	4	44.4%
Yes	5	55.6%
Response criteria		
Partial remission	5	55.6%
Complete remission	4	44.4%

All patients in this relapse group had received IV steroids as first-line therapy for a duration ranging from 15 to 20 days with a mean of 15.89 (± 1.83) days. Ongoing hemolysis after the first line of treatment was reported in 4 (44.4%) patients who received adjuvant therapy (IVIG). Two (22.2%) patients additionally needed plasmapheresis to achieve control of their hemolysis.

### Treatment of relapses

IV steroids were used in 5 (10%) patients, 2 (4%) patients resumed full dose oral steroids with mycophenolic acid, 1 (2%) patient started mycophenolate mofetil only, and another patient (2%) required IV steroids + azathioprine. Regarding maintenance treatment after the relapse, 6 (12%) received full dose oral steroids + mycophenolate mofetil, one (2%) patient resumed full dose oral steroids + azathioprine, one (2%) patient started only mycophenolate mofetil, and one (2%) just resumed to full dose oral steroids.

## Discussion

The study group comprised 30 (60%) females and 20 (40%) males. Their age ranged from 1.1 to 14 years, with a median of 8.25 years. These findings are consistent with the evidence of literature highlighting a higher incidence of autoimmune diseases among females with a higher prevalence of AIHA among females [3, 21].

However, these findings are inconsistent with the Mayo Clinic experience, highlighting a higher incidence of AIHA among males, 65.7% [2]. The largest cohort of pediatric AIHA patients from France showed that males were predominant over females in all study groups (AIHA alone, AIHA/Evan’s syndrome, and total cohort) [22].

In our study, the median age at diagnosis (IQR) was 5.5 (2–10) years with a range of 0.9–12 years which is approximately similar to Weli et al., which was 4.5 years [23].

In the present study, 42% of cases were of children of consanguineous marriage, and only two patients (4%) had a positive family history of autoimmune disease. These findings are similar to ones reported by Aladjidi et al. when 8% of the included patients had a positive family history of malignancies and autoimmune illnesses [22].

The primary distinction in childhood AIHA is the possibility of developing Evans syndrome (ES). ES was first classified as an autoimmune illness in which AIHA, immune thrombocytopenia, and/or immune neutropenia develop concurrently or sequentially without any underlying cause. ES has been reported to occur in 13–73% of children with AIHA [24-26].

In the current study, 12 (24%) patients have associated thrombocytopenia and were diagnosed with Evans syndrome. This is lower than reported by Tantawy et al., who stated that Evans syndrome was reported in 50% of cases [3].

In the present work, 46 (92%) of cases had Coombs test positive, and the rest of the cases, 4 (8%), showed negative tests. These findings are consistent with a published report that revealed 11% of AIHA patients showed negative Coombs tests [27, 28].

In the current study, 40% had primary AIHA versus 60% had secondary AIHA. These findings are similar to many reports, which indicated the prevalence of idiopathic or primary AIHA approaching 40% compared to secondary AIHA [22].

In the current study, patients diagnosed with secondary AIHA to autoimmune diseases such as SLE were 24%, those secondary to drugs with autoimmune disease were 4%, and those secondary to viral infection with autoimmune disease were 8%. These findings are consistent with many studies in the literature which demonstrated that secondary AIHA is most frequently caused by autoimmune illnesses such as SLE and juvenile idiopathic arthritis, and infections, generally viral in nature [10]. In our study, there were no cases of underlying malignancy [2].

Transfusion is a key player in the management of AIHA; it is frequently indicated for symptomatic or fast-progressing anemia, leading to life-threatening status, but the limitations of blood transfusion are mainly transfusion reactions and recurrent mismatch [13].

Our data showed that blood transfusion was required in all patients with a median transfusion frequency of 2 (IQR: 1–2; range 1–8) units/week. The recurrent mismatch was found in 44 (88%) cases. These findings are markedly higher than those reported by Zhu et al., who reported the rate of repeated ABO mismatch by 31.6; in addition, all cohorts were highly reactive for the indirect agglutination test [29]. Others reported 12–40% of transfused patients developed clinically significant alloantibodies inducing rapid hemolysis and causing hemolytic transfusion reactions [30, 31].

In the current study, IV steroids were used as a first-line treatment among the included patients. Thirty-eight (76%) cases responded, while 12 (24%) were non-responders. They were rescued, and the treatment for non-responders was IVIG used in 11 (22%) as adjuvant therapy or plasmapheresis applied in 5 (10%) cases. These findings are consistent with international guidelines, confirming a response rate of 80% for steroids as a first line in treating AIHA among pediatrics [10, 32]. Our findings are also similar to those reported by Tantawy et al., as the response rate for steroids was 94% [3].

We found that secondary AIHA showed female sex predominance while primary AIHA patients were predominantly male. Our children with secondary AIHA were older at the presentation. These findings are consistent with the cohort presented by Naithani et al., who reported a higher prevalence of secondary AIHA among females who were older at presentation with a median age of 11 years (range 0.2–17) [33]. On the contrary, the data published from other centers reported a younger age at presentation, with a median age of 2 years [34] and 8 years [35].

At the onset, all patients with primary AIHA had jaundice and dark urine, while jaundice was seen in 60% and dark urine in 70% of the secondary group (*p* = 0.001 and 0.007, respectively). These findings are consistent with the study conducted by Naithani et al. who reported that jaundice was much more prevalent in primary cases (70%) than in secondary (44%) [33].

Our results found that relapse occurred in 9 cases, 2 cases with 1^ry^ AIHA, and seven patients with secondary AIHA. However, Naithani et al., in 2007, showed relapse in 4 patients only; all belonged to the idiopathic (primary) group [33].

In the current study, 41 (82%) patients showed improvement during the withdrawal of steroids, while 9 (18%) patients relapsed during the withdrawal of steroids. These findings are consistent with the study conducted by Dussadee et al., who reported relapse rates among children with AIHA were significantly higher among patients with a shorter period of steroid tapering [36]. Moreover, Mayo Clinic reported a relapse rate of 22.8% among a cohort of 35 AIHA patients [2].
